# Comprehensive assessment of snow leopard distribution and population in the Indian Trans-Himalaya, Ladakh: Standardizing methods for evidence-based conservation

**DOI:** 10.1371/journal.pone.0322136

**Published:** 2025-05-07

**Authors:** Pankaj Raina, Ninad Avinash Mungi, Ujjwal Kumar, Aman Deep Rathi, Niazul H. Khan, Dimpi A. Patel, Anchal Bhasin, Shikha Bisht, Lex Hiby, Bivash Pandav, Mohd. Sajid Sultan, Jigmet Jigmet Takpa, Yadvendradev V. Jhala

**Affiliations:** 1 Department of Wildlife Protection, Leh, Union Territory of Ladakh, India; 2 Wildlife Institute of India, Dehradun, India; 3 Center for Ecological Dynamics in a Novel Biosphere (ECONOVO), Department of Biology, Aarhus University, Aarhus C, Denmark; 4 Tiger Cell, National Tiger Conservation Authority, New Delhi, India; 5 Conservation Research, Cambridge, United Kingdom; 6 Indian National Science Academy, Hosted at National Centre for Biological Sciences, Bangalore, India; Amity University, INDIA

## Abstract

Effective conservation of threatened species depends on accurate scientific assessment of their occurrence and population status. This information is often lacking or has poor scientific reliability for low-density carnivores, such as snow leopards (*Panthera uncia*) that inhabit remote and challenging habitats. We address prevalent sampling and study design limitations and evaluate the population and distribution of snow leopards and their prey using a double sampling approach across the Trans-Himalayan Ladakh (~59,000 km^2^), India. We used spatial data on the sign occurrence of snow leopards, collected by replicate sign surveys of 6,149 km to model occupancy and potential distribution. Regions representing varying occupancy were used to stratify density-estimation using spatially explicit capture-recapture by sampling 956 camera trap locations with an effort of 97,313 trap nights. Camera traps captured 26,130 images of 126 unique snow leopards identified by a pattern recognition program using their distinctive forehead pelage patterns. Low-elevation grassy and resource-rich regions, with moderate climatic conditions and complex terrain had higher presence of herbivores and consequently higher occupancy of snow leopards. Density of snow leopards was estimated at ~ 1 per 100 km^2^ with a large movement parameter (σ) of 4.09 (SE 0.15) km and detection at home-range centre (g_0_) of 0.003 (SE 0.0003). Snow leopard density reached up to 3.18 per 100 km^2^ and was driven by the distribution of their wild and domestic prey in suitable habitats. The snow leopards in Ladakh occupied 47,572 km^2^, holding globally highest extensive densities of snow leopards in Hemis National Park (2.073 ± 0.278 per 100 km²), Kargil (1.257 ± 0.480 per 100 km²), and Leh (1.029 ± 0.434 per 100 km²), and making one of the world’s largest contiguous populations of 477 (CI 380–598) snow leopards. This population holds global significance as an important source of snow leopards, predominantly (61%) occurring in multi-use areas and closely linked with wild and domestic herbivores. We offer a robust and comprehensive method for large-scale population estimation of snow leopards, applicable globally. The co-occurrence of humans and wildlife across the landscape underscores the need for inclusive and evidence-based conservation planning, especially considering the impending large-scale infrastructural development and escalating global climatic changes.

## 1. Introduction

Large carnivores are among the most iconic species, symbolising a thriving ecosystem and conservation optimism [1]. Their apex position in the food chain is linked to cascading positive impacts on trophic regulation, nutrient cycling, and disease regulation [2,3]. And yet, large carnivores are among the widely extirpated species, leading to enduring impacts on the functioning of our modern ecosystems [4]. Several global initiatives and regional policies have been conceived to conserve and recover the remaining large carnivore species [5]. Although rare, bright spots for carnivore conservation and recovery are unfolding worldwide [6,7]. A vital facet of such successful programs is the implementation of scientific population monitoring to understand population trends and the underlying drivers that assists in prioritizing scale-sensitive investments [8,9]. However, population monitoring faces considerable challenges due to the elusive nature of these species, challenging habitat conditions, limited scientific methodologies, and financial constraints. These challenges are particularly exacerbated in the Global South, which is home to a disproportionately rich diversity of large carnivores, demanding innovative solutions [4].

Among the enigmatic large carnivores, the snow leopard (*Panthera uncia*) is a flagship species in the mountainous regions of South and Central Asia, spanning 12 countries [10]. Snow leopards are known for their elusive and large-ranging behaviour, necessitating a landscape approach for population monitoring and conservation [11]. Conversely, most population assessments have been conducted over smaller areas, usually high-density regions, and then extrapolated to larger scales, potentially leading to inflated population estimates [12]. Moreover, unlike standardised population assessment methods used for other large carnivores such as tigers and leopards [13], snow leopard occurrences are often inferred from opinion-based surveys, which have been shown to potentially undermine population and distribution parameters [14]. A very limited region of the snow leopard range has peer-reviewed population estimates [12]; therefore, a global estimation of the snow leopard population is largely speculative. It seems likely that inflated local estimates of snow leopard populations have led to their downlisting from Endangered to Vulnerable in 2017, a decision that faced criticism due to its potential for scientific misinterpretation [15]. With an estimated 221–450 snow leopards poached annually worldwide [16] to primarily fuel the illegal trade of their pelts, downlisting can have far-reaching ramifications for their conservation. Additionally, ongoing climatic changes present complex challenges for snow leopards and their ecosystems. There is an urgent need to correct for the inherent limitations of population and distribution assessments by large-scale implementation of robust scientific methods. Importantly, the population assessment must be repeatable and sensitive to monitor trends in population parameters due to poaching or global changes, which can further inform the decisions on downlisting/listing the global conservation status of the species. This will also help relate population parameters to changing climate, land-use systems, and prey availability to timely prioritize conservation investments. Methods currently used for population assessments often include data from multiple years, undermining recognition of risks to population and hindering preventive/mitigative actions [12,15]. Such misrepresentations have obscured recognition of extinctions and timely actions for large carnivores in the past [17], and hence must be avoided in the case of snow leopards. Current population assessment methods for snow leopard are not sensitive to recognize patterns of population trends, necessitating development of a robust repeatable method that can be applied globally, without compromising the sensitivity to detect changes.

Large-scale robust population assessments can be effectively developed by integrating technological advancements and scientific methods. Technology such as camera traps and mobile applications has been widely used to ensure effective sampling and data transparency for reliable population assessments [18,19]. Advancements in pattern recognition, big-data analysis, and robust statistical packages have facilitated the development of scientific protocols for accurate and precise population estimation [9]. It is imperative to harness these advancements within the double-sampling framework, where snow leopard occupancy is initially sampled across the landscape, followed by a systematic sub-sampling of densities across the occurrence gradient. This sampling scheme has become a standardized approach for national-scale population assessments of tigers and leopards in snow leopard range countries like India and Nepal [13,20], but has been oddly limited in case of snow leopard assessments. A transnational collaboration for the Population Assessment of World Snow Leopards (PAWS) was endorsed during the Bishkek Declaration in 2017 to target robust population estimation at national and global levels [21]. Suggestions from the PAWS and other successful large-scale population assessments underscore the efficacy of utilising technological innovations, robust scientific protocols, and a double-sampling approach [14,22–24]. A challenge to globally adopt a single design for evaluating population and distribution of snow leopard was the unavailability of a sensitive and proven robust estimation at a large-scale, representing diverse habitats occupied by the species. Hence, PAWS suggestions were seemingly compelled to allow contextual flexibility in estimation methods to accommodate spatial and logistic constraints, so as to make it applicable for large-scale implementation.

While recent population estimations have been noted to subscribe to the double-sampling design suggested by PAWS, the methods and sampling designs vary to the extent that can render estimates non-comparable or unreliable in understanding long-term population trends. Key limitations amongst existing assessments could be categorised into 1) biased presence indicators: use of only opinion-based estimation (e.g., using only interview surveys for deriving snow leopard occurrence) [14], or using methods less sensitive to record the diversity of presence signs (e.g., use of a relatively less-sensitive vehicular survey to record signs) [25]; 2) sampling biased towards small areas of high-density population or homogenous landscape units (e.g., productive areas in river valleys) [12]; 3) low-intensity sampling within extremely large sampling units (e.g., one camera trap/sign survey within 15 × 15 km grids) [25]; 4) violation of population closure in camera trapping (e.g., pooling data sampled over several years as a single-period sampling); and 5) misidentification of snow leopard individuals due to distortions caused by thick coats in their pelage patterns [26]. Addressing these limitations and achieving accuracy and precision in population assessments is essential for monitoring population changes resulting from factors such as poaching, anthropogenic development, and climatic change. While PAWS has provided a vital guideline for streamlining global efforts on the assessment of snow leopard population and distribution, it further requires field translation to experimentally demonstrate a reliable design that can be globally upscaled, by addressing the present prevalent limitations.

In the present study, we developed and implemented the largest known intensive snow leopard assessment across the union territory of Ladakh in India. We implement a double-sampling approach across a 59,150 km^2^ landscape, using occupancy surveys, distribution models, and spatially explicit mark-recapture using camera traps. Our primary goal was to assess the distribution and population of snow leopards and their prey in Ladakh and demonstrate a robust approach for designing a sensitive assessment applicable at a multi-landscape scale. We utilized the tracking skills and high-altitude endurance of local communities, cutting-edge technology, and integrated the administrative set-up in our surveys to ensure the acceptance of our results for conservation management of snow leopard ecosystems and policy uptakes.

### 1.1 Study area

**Fig 1:**
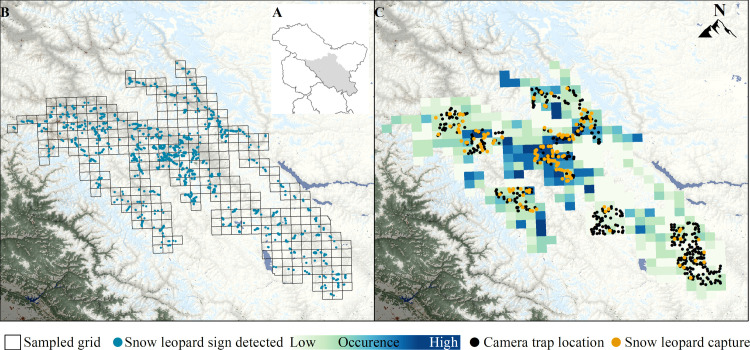
Study area and sampling design. A) Location of the study area in the Union Territory of Ladakh, India. B) Location of occupancy sampling grids (10 × 10 km) across the study area, where every grid had multiple sign surveys to record snow leopard signs. C) Locations of camera traps and cameras with snow leopard photo-captures.

We sampled the largest contiguous snow leopard habitat in India, located in the union territory of Ladakh [10]. Situated in the northernmost region of India, Ladakh’s biogeography is characterized by a trans-Himalayan landscape, with its signature high mountainous and cold desert conditions [27] (Fig 1). The landscape witnesses a west-to-east gradient in decreasing above-ground productivity with increasing cold aridity. Vegetation is predominantly dry alpine steppe consisting of widely spaced shrubs in sparse grassy areas. Valleys are generally moister and dominated by relatively higher plant cover. The vegetation diversity supports eight wild large herbivore species wild ass (*Equus kiang*), argali (*Ovis ammon*), wild yak (*Bos mutus*), Tibetan antelope (*Pantholops hodgsonii*), Tibetan gazelle (*Procapra picticaudata*), bharal/blue sheep (*Pseudois nayaur*), Asiatic ibex (*Capra ibex sibiricap*), and Ladakh urial (*Ovis vignei vignei*); the last three being the preferred prey of the snow leopard [28]. Besides snow leopards, other large carnivores found in the region were the Himalayan brown bear (*Ursus arctos isabellinus*), Himalayan wolf (*Canis lupus himalayensis*), and Eurasian lynx (*Lynx lynx*). Snow leopards also predate on domestic livestock (domestic yak calves, dzo, cattle, ponies, mules, sheep, and goats) in the region, which is a major cause of conflict with local communities [29–31]. Local communities have adapted to live with higher interactions with wild animals while procuring irreplaceable ecosystem services for livelihoods and cultures. Land use is witnessing rapid changes with large investments in modern infrastructure, the tourism sector, agriculture, and livestock industry (Vision 2050 for Ladakh UT 2020). The cultural reverence and tolerance for wildlife interaction juxtaposed with the region’s economic development marks a unique human-wildlife relationship in the region [32–34].

## 2. Methods

We used a phased double-sampling approach for estimating landscape-level distribution and population. During phase I, we estimated the occupancy of snow leopards and their prey species using sign surveys and scan sampling. We used this information to spatially model the distribution of snow leopards and their prey species across the region. In phase II, we sampled representative subsets across the occupancy spectrum, representing varying density levels, using camera traps to estimate the density and abundance of snow leopards in the region.

### 2.1 Data collection

Ground sampling data for Phase I was recorded using ‘MSTrIPES’ mobile application customised for the snow leopard population assessment [35]. This open-access mobile app automatically records the spatial and temporal attributes of occupancy trails and plots, along with geotagged photographic evidence of sign detections. The app can be accessed in English or in 10 other local Indian languages. Information recorded via the app was transferred onto desktop software customised for data archiving and structuring. This ensures accurate and easy data entry, transparency, and long-term archiving in the digital infrastructure. The sampling was conducted in collaboration with local community enthusiasts, naturalists, and Wildlife Protection Department staff and researchers, totalling approximately 30,000 working-tenure days (“man-days”) over two years. Along with information on snow leopard population, the teams also conducted simultaneous assessments on wild herbivores population, vegetation communities, wildlife movement, habitat-use, and land-use mapping. This enabled the program to become holistic towards ecosystem monitoring, while reducing the extra-cost. The total incurred cost for the study was about USD 183,000 (INR 16,000,000), which includes the cost of equipment, salaries of researchers, and other field expenditures such as travel, lodging, food, etc. Apart from this, wildlife departmental staff and equipment available to the department, such as camera traps, handheld GPS, and binoculars, were also fully utilized for the fieldwork. To streamline consistency in field data collections and ensure effective utilization of diverse skills necessary in the field, the members were trained on sign/animal identification, vegetation identification, use of mobile applications, camera trapping, and animal tracking via multiple workshops. Subject experts conducted a trial field sampling to ensure consistency in field methods and train other team members for uniform data collection. We ensured that every sampling team was comprised of experts on these different techniques. An illustrated field guide was developed and shared to ensure cohesive field sampling. Moreover, every sign was photographed through mobile applications, which experts could subsequently confirm.

#### 2.1.1 Occupancy survey.

The landscape was divided into a 10 × 10 km grid, as it represented the average home range of snow leopards in the region (Raina et al., unpublished telemetry data of 10 snow leopards), thus an ecologically relevant scale for making distribution inferences. To systematically distribute our sampling spatially within every grid cell to cover inherent habitat heterogeneity [36] (Fig 1A), it was further divided into sub-grids of 5 × 5 km. Within every cell of such a 25 km^2^ sub-grid, we assessed carnivore occupancy, herbivore occurrence, and human impacts. For carnivore occupancy, at least one sign-search survey of approximately 5 km each was conducted to record signs of the targeted carnivore species (e.g., snow leopard, wolf, lynx, fox, etc.) in every sub-grid cell; thus, having a minimum of 4 independent spatial replicates in the sampling grid cell. Animal trails, ridgelines, and water gullies were targeted to maximise the chances of encountering signs. Signs such as pugmarks, scats, and scrape marks that could be assigned to a species were recorded. A pictorial guide for identifying the signs of key species was provided to the sampling team during the training sessions. For herbivore occurrence, a point transect of variable length was sampled every 500 m on the occupancy trail to record the sightings of herbivores. At every plot, the region was scanned for at least 5 minutes to detect herbivores within 360^o^ view using a calibrated binocular (Celestron 71422 Cavalry) to measure the herbivores’ distance and bearing from the sampling point. Species, group size, distance, bearing, detection time, and plot location were recorded upon detection. Subsequently, using GIS hill-view analysis and effective sighting overlap we removed sightings of potentially similar animal groups, by retaining the observation with largest animal count. To sample human impacts on the habitat in the region, we recorded signs by counting livestock tracks, recording the presence of vegetation harvest on the plot, documenting the number and condition of livestock corrals, counting vehicle tracks, recording the frequency and number of people seen, along the occupancy trails. We effectively sampled 351 grids covering 35,100 km^2^ of habitat area by walking approximately 6149 km of trails across the landscape (Fig 1A).

#### 2.1.2 Population estimation.

Phase II of the double-sampling approach consisted of extensive high-density camera trapping and subsequent identification of snow leopard individuals to estimate their population in a spatially explicit mark recapture framework [37]. Camera trapping was done in seven blocks representing high-occupancy areas (Hemis, Kargil), moderate occupancy areas (Nubra, Leh), and low occupancy areas (Changthang 1, Changthang 2, and Zanskar), with each block being >900 km^2^. The blocks were unbiased with respect to the probability of snow leopard occurrence (Fig 1B) and were dictated by the logistical constraints of sampling at this high-altitude rugged terrain. For spatially covering the area of each block without “holes” and deploying multiple cameras within the home range of every snow leopard we used a 3 × 3 km grid to guide the systematic deployment of camera traps. Within each of these 3 × 3 km grids, camera traps were deployed to maximise snow leopard capture based on signs (e.g., scat, pugmarks, kills, etc.) along the trails identified during the occupancy survey. We used motion and thermal detection cameras (Reconyx Hyperfire 2 IR), which were tested for operation at low ambient temperatures. Camera traps were positioned to capture the forehead of the snow leopards. We used scent lure that had only localized effect in making the snow leopard to bend down and expose its forehead to the appropriately positioned camera trap. Evidence from studies on other large carnivores suggests no bias in density estimates when the trap locations within the study area are baited or lured [38,39]. Camera traps were kept active for a maximum of 125 days in a block. We effectively sampled 8,604 km^2^ in seven blocks using 956 camera trap locations and 97,313 trap nights (Fig 1B).

**Fig 2:**
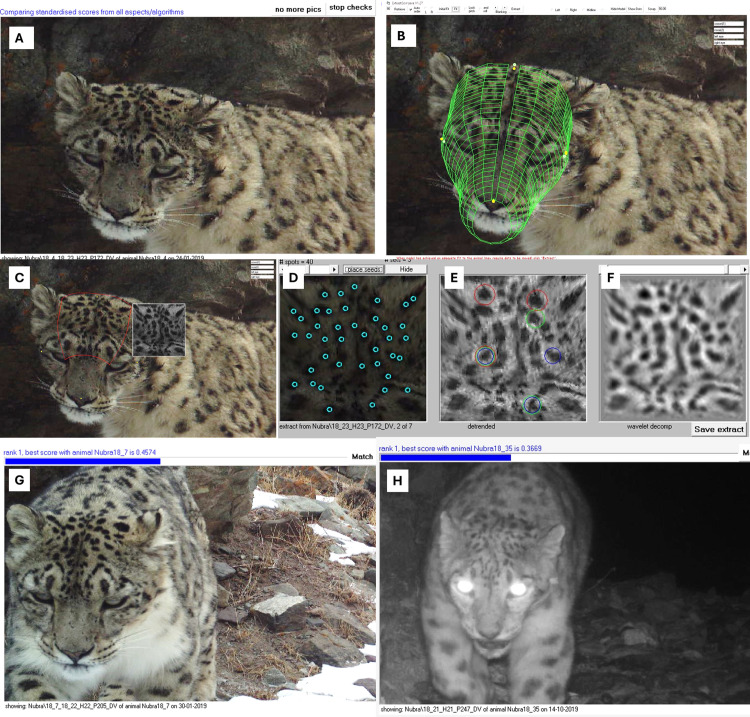
Identification of snow leopard individuals. Process for identifying snow leopard individuals using ExtractCompare software. A) Strategically placed camera traps allow for a photo-captures of the forehead of snow leopards. B) Reference points are placed on the crown, eyes, and nose to permit fitting a 3D model on the forehead to correct for the varying angles at which the photo may have been taken. C) After correcting for the angle, the forehead pelage pattern is extracted as a 2D image. D-F) Reference cues are generated to digitize the pelage pattern and saved quantitatively as unique data per individual photograph, forming a library. G) The software finds the best possible matches of the forehead pattern from the library and ranks in order of the similarity index. Independent expert observers subsequently use the similarity index to verify the patterns and confirm same individuals (G) from other individuals (H).

Every camera trap photograph was geotagged and stamped with the time and date information in the metadata. The photo-captured species were identified using an AI-based software program, CaTRAT, customised to identify Himalayan species [40]. The identified species were subsequently validated by biologists. Snow leopard photographs were obtained from this data archive. We used a customised pattern recognition program within Extract-Compare [41] to digitise and archive the pelage pattern of the forehead for each usable snow leopard photograph. We identified individual snow leopards through their unique forehead pattern. The identification based only through pelage patterns on flanks has limited reliability due to the thick and long body fur which can get ruffled by the wind causing the pattern to look different for the same individual in different photo-captures, while the forehead fur is shorter and the pelage patterns unlikely to change [42]. Previous studies implying use of forehead patterns had placed camera traps for detecting flank pattern and resultantly obtained lesser proportion and low-quality images of forehead [42]. We resolve the challenge in reliable identification of unique snow leopards by study design and objective pattern recognition, which was never tested or utilized at a large scale. The pattern recognition program fits a three-dimensional model to the forehead of snow leopard photographs, extracts patterns, and develops a unique key based on these patterns (Fig 2). The program then compares these patterns across the archived database to show probable matches with a similarity score to determine whether the photos belong to the same individual leopard. Subsequently, a set of trained biologists verify the photograph matched for 100% verification, using minimum two rounds of independent verifications.

#### 2.1.3 Environmental variables.

We used a suite of ecologically relevant sampled variables and remotely sensed datasets on climate, terrain, vegetation, water, and disturbance factors, which are important for determining species occurrence. All variables had a resolution of less than 1 km and were rescaled to 1 km resolution in ArcGIS 10.6.1. The choice of these site covariates was based on the ecological requirements of prey species and snow leopards. Ground sampling data collected along occupancy trails were used to derive the sign encounter rate of human disturbance indicators, livestock occurrence, and herbivore occurrence in the sampled region. Since climate is a major determinant of herbivore and carnivore distribution patterns [43,44], we used maximum temperature of summer, minimum temperature of winter, annual precipitation, precipitation during the driest and wettest season for modelling their occurrence [45]. As herbivore species respond differentially to forage availability, elevation, and ruggedness of the terrain [25,46], we therefore used elevation, slope, proportion of valleys, terrain ruggedness, dry grass dominated areas, moist grass dominated areas, and areas under natural open ecosystems mapped from LANDSAT satellite data [47] to index terrain characteristics for species distribution models. To represent seasonal changes in vegetation cover of an area, we used difference between Enhanced Vegetation Index (EVI) of wet season (June, July, and August) and dry seasons (December, January, and February) derived by averaging monthly MODIS composite for the year 2018 [48]. As water is an important resource in desert ecosystems, we used proximity to water sources mapped from LANDSAT satellite data [47]. As large wild species often need areas that are free from intensive human modifications [49,50], or permanent snowy areas, we used proportion of built up area and snowy areas mapped from LANDSAT satellite data [47]. The source, details and resolution information of all covariates used for modelling species distributions are listed in S1 Table. Thereafter, we developed a Pearson’s correlation matrix between all variables (S2 Table) and included only non-correlated variables (-0.5 < r < 0.5), and in case of correlated variables, one with more direct ecological relevance was used.

#### 2.1.4 Ethics statement.

The study was conducted with permission and participation from the Department of Wildlife Protection, Leh and the Ministry of Environment, Forests, and Climate Change, Government of India under the provisions of the Wildlife (Protection) Act, 1972. During the camera trapping survey, villagers were informed about the placement of cameras and the intended use of wildlife photographs.

### 2.2 Analyses

#### 2.2.1 Occupancy analysis.

The extent of habitat occupied by a species is often used as one of the state parameters for evaluating its status. Detection of a species or its signs is inferred as an area being occupied, whereas non-detection could either result from the absence of the species or failure to detect the species. Occupancy modelling uses capture frequencies across multiple surveys to compute detection probabilities and correct for imperfect detection. We used a likelihood-based approach to estimate detection probability and occupancy using PRESENCE Software (Version 13.10) [36]. The grid of 10 × 10 km was considered for deriving occupancy, as the scale was ecologically relevant. Every carnivore occupancy survey within the grid was considered as a single replicate, where sign detection was considered as presence, and absence of sign as non-detection or possible absence. We further used variables representing ecological site characteristics explaining hypothesized relationship with the snow leopard occupancy (S1 Table) validated using exploratory analyses.

We developed three sets of models: naïve occupancy, detection-corrected occupancy, and detection-corrected covariate-based occupancy. Foremost, we derived ‘naïve occupancy’ by summing the habitat area of all grids where snow leopards were detected. Detection-corrected occupancy was derived by correcting naïve occupancy by detection probability estimated using spatial replicates. Subsequently, occupancy was modelled using site variables and detection modelled using spatial replicates and survey covariates. Based on the *a priori* explored ecological hypotheses (S1 Table), different covariate-based occupancy models were computed. The model with the least AIC was considered the best model explaining the snow leopard occupancy. In case when the best model differed from subsequent models by less than a difference of 5 AICs, we averaged the model parameter estimates and statistics of all such competing models using their relative weights. Occupancy analysis provided the likely distribution of snow leopards in the sampled region, as well as its ecological determinants. A robust relationship established between site covariates and snow leopard occupancy would permit extrapolation of snow leopard occurrence across the unsampled grid cells of the landscape wherein the covariate information was available [36].

#### 2.2.2 Species distribution modelling.

As snow leopards and their major prey (blue sheep, ibex and urial) could potentially occur in regions that were not sampled in Phase I, we modelled the potential distribution of snow leopards and their prey across the Ladakh region using Maximum Entropy (MaxEnt) [51]. MaxEnt is one of the most widely used algorithms for species distribution modelling, which calibrates environmental variables from species presence locations and compares them with a set of random background locations from the study area. Being a presence-background model the extent of the background environment influences the species-environment relationship [52], which necessitates appropriate ecological criteria to define the background. Since we only consider natural habitats for this study, we used a reliable land use land cover map [47] to remove areas like infrastructure and settlements, water bodies, and regions of permafrost (above 5000 m). To address the autocorrelation within presence points, we spatially thinned the presence locations at 1 km scale, so that every pixel (1 km^2^) will have only one presence location. Even though sampling was systematically distributed using a grid, the intensity of sampling in a grid may vary (Fig 1). To account for this variability, we used bias correction by including a sampling density map. For deriving this map, we used sampled plot density within every 25 km^2^ grid to generate 2D kernel density surface. MaxEnt uses this bias file to correct for the variability contributed by sampling bias. For assessing model performance, we divided the presence data for each species into training (80%) and testing (20%) sets, for MaxEnt modelling and independent accuracy assessment, respectively. This was done using k-fold (k = 5) partitioning with proportionate samples across elevational gradient, where we used each k-partitioned set, for every species, for MaxEnt models.

We used the partitioned presence locations for every species and developed habitat suitability models using linear, quadratic, hinge, and product features each with 100 bootstrap simulations. We performed Jackknife simulations (n = 100) to estimate the contribution and effects of different covariates on species habitat suitability and reported the results as a logistic output in MaxEnt [51]. For every species, results of all the k-partitioned model runs were averaged, which provided a probability of potential distribution for every pixel that varied from 0 to 1. All pixels greater than a threshold that yield highest True Skill Statistics (TSS), were considered potentially suitable for species occurrence. To assess the TSS, we used the 20% test data that was not used to build the model and the background data used by MaxEnt. This TSS was used as the primary index of accuracy for the estimated species distributions [51]. The averaged Area Under the Receiver Operator Characteristic Curve (AUC) of MaxEnt model was considered as the explanatory power of the model.

For deriving a consolidated prey prevalence index, we used potentially suitable pixels for all herbivore species that were above the highest TSS value. The probabilities per pixel for all herbivores were then spatially summed to derive the prey prevalence index, which was used along with other variables for modelling snow-leopard distribution. The estimated snow-leopard occurrence map was used as a habitat mask for population estimation analyses as explained below. In our case, the advantage of using MaxEnt model instead of occupancy model for population estimation was that it could extrapolate species occurrences beyond the sampled grids effectively modelling species occurrence for the entire study region. Nonetheless, to verify the utility of MaxEnt for modelling snow leopard distribution, we compared the occupancy probability of a grid cell with the average MaxEnt probability in the cell, across the sampled region. We assume a logarithmic trend where occupancy probability increases with initial increase in MaxEnt probability and subsequently remains constant around 1. An agreement would indicate the utility of MaxEnt in effectively modelling the species occurrence.

#### 2.2.3 Population estimation.

After identifying individuals and their capture histories across camera traps, we estimated snow leopard density using maximum likelihood based spatially explicit capture recapture models (SECR) [24]. The advancement of spatial capture-recapture techniques [24] has significantly enhanced the accuracy and precision of density estimation by incorporating the spatial location of animal photo-captures, and suitable covariates for modelling abundance across the landscape simultaneously [13,39]. Variability in probability of photo-capturing individuals may arise due to location of camera traps relative to the activity centre of individuals and their heterogenous movements [24]. SECR model accounts for these variabilities. The detection process is represented by a mathematical function that describes an animal’s declining probability of being detected away from its activity centre. A simple detection function has two parameters, I) g_0_: detection probability at the activity centre of the animal, and II) σ: spatial scale of detection that declines with distance from the activity centre. We model this decline using half normal detection functions.

We could not reliably identify sex of many of the photographed individuals due to the thick fur of snow leopards that covers the genitals. Hence, sex-specific heterogeneity could not be accounted. We formulated three models 1) landscape null model: We pooled capture histories of the seven sampling sites in one session for robust parameter estimates. The arising density represented variation across the population gradient and was hence assumed to vary constantly across the landscape habitat mask (D ~ 1); 2) session-specific null model: densities were derived for every sampling block (n = 7) and the pooled density estimate was applied across the entire habitat mask area in the landscape; 3) session-specific covariate model: single session model used including the heterogeneity of all sampling blocks and modelled using site covariate. We used MaxEnt derived occurrence probability as a covariate to model the density across the suitable habitat mask of snow leopard. The AICc for models with similar parameters (model 1 and 3) were compared to select the model with least AICc and predict population for Ladakh.

We conducted analysis in R program environment with package secr 4.6 [37]. Subsequently, the density surface was superimposed with protected area boundaries to understand the population with respect to current protection regime and identify hotspots for prioritizing conservation investments. Individuals using protected areas although with their activity centres outside, were estimated using the buffer of double σ around every protected area.

## 3. Results

**Fig 3:**
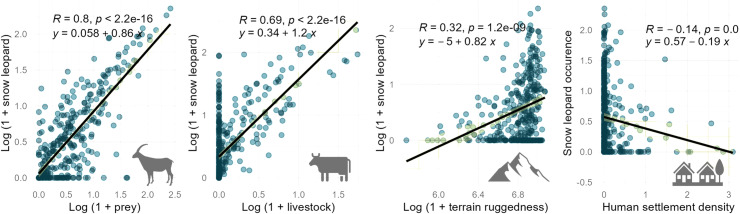
Relationships between snow leopard and environmental variables. Snow leopard sign encounter rates significantly increased with A) increasing wild prey encounter rates, B) increasing livestock encounter rates, C) increasing terrain ruggedness, and D) decreasing human settlement density. These graphs were used to strengthen the choice of variables in distribution modelling.

We found a total of 9525 signs of snow leopards across the landscape (Fig 1A). We also recorded 407 observations of blue sheep, 155 observations of urial, and 226 observations of ibex from across the region and used these for the MaxEnt model. Exploratory analyses revealed positive and significant relationships between increasing snow leopard sign encounter rate with increasing wild prey encounter rate, increasing livestock encounter rate, increasing terrain ruggedness and decreasing human settlement density (Fig 3). This strengthened our a priori hypotheses and supported using these variables in modelling occurrence of snow leopard in the region.

### 3.1 Occupancy modelling

**Fig 4:**
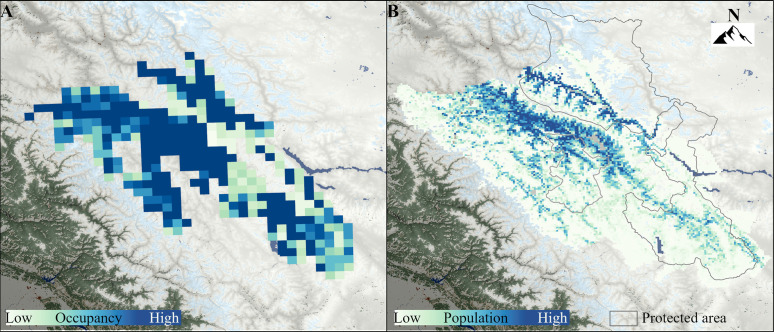
Snow leopard distribution and density. A) Occupancy of snow leopard in the sampled region of Ladakh. B) Population density of snow leopard in Ladakh.

The naïve occupancy of snow leopard in the region was 23,000 km^2^ (65.5%). The detection corrected null occupancy was 25,709 km^2^ (73.2%; CI: 25,201–26,263), whereas covariate-modelled detection corrected occupancy was 25,308 km^2^ (72%; CI: 22,726–28,091) (Fig 4A). The best amongst competing models (S3 Table), was the covariate model having positive contribution by ungulate encounter rate (large wild herbivores + domestic herbivores), whereas elevation and human settlement had negative coefficients (Table 1). All grid cells were modelled to be occupied by snow leopard, with higher occupancy in and around Hemis National Park and Nubra Shyok Wildlife Sanctuary.

**Table 1:** Snow leopard occupancy model. Model parameter estimates snow leopard occupancy (Ψ) and detection (P) in Ladakh The sign and magnitude of the ß estimate provides the relative influence of the ecological variables on snow leopard occupancy.

Parameter	Variable	ß Estimate	SE
Ψ	Intercept	3.99	0.8
Ψ	Ungulate encounter rate	9.34	2.19
Ψ	Elevation	-1.12	0.25
Ψ	Human settlement	-0.64	0.22
P	Detection – Intercept	-0.04	0.07

### 3.2 Species distribution models

**Fig 5:**
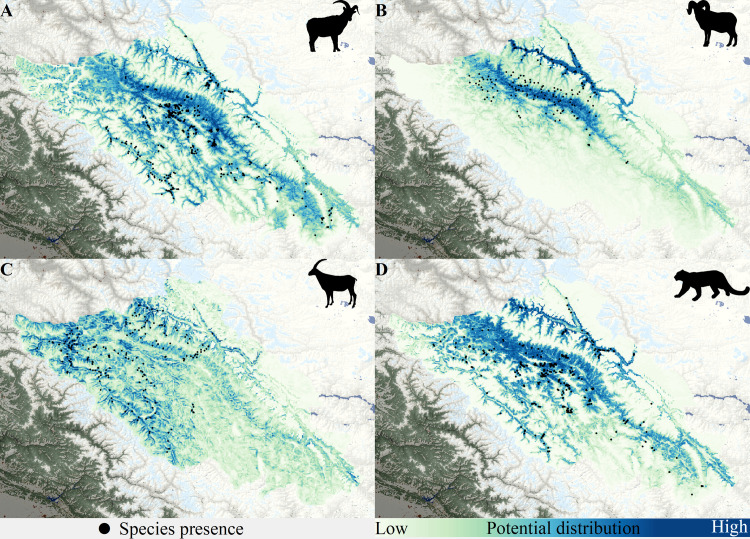
Distribution of snow leopard and prey species. Potential distribution derived using MaxEnt for bharal/blue sheep *Pseudois nayaur* (A), urial *Ovis vignei* (B), ibex *Capra sibirica* (C) and snow leopard *Panthera uncia* (D) in Ladakh.

MaxEnt modelling highlighted differential contribution of ecological covariates, in consonance of our a priori hypothesis (S1 Table), in estimating the distribution of wild herbivores. Bharal presence was positively related with mid-elevation areas with moderate moisture and moderate vegetation cover (S1 Fig). Urial presence was positively related with low elevation valleys with warmer temperatures and geographically around the Indus River catchment in the hilly regions (S1 Fig). Ibex presence was positively related with higher slope in moderate elevation areas in the moist regime of the landscape with higher grassy cover (S1 Fig). These wild herbivores largely covered the entire landscape, with stronger individual geographic affinity (Fig 5).

MaxEnt modelling of snow leopard presence highlighted a strong positive influence of wild prey occurrence (model contribution 95%). Other variables influencing snow leopards were intermediate elevation, moderate ruggedness, and areas with less permanent snow cover (S1 Fig). The estimated occurrence of snow leopard in the landscape was 47,572 km^2^ (Fig 4). The modelling had reliable accuracy with TSS = 0.79, and AUC = 0.86 (SD 0.001).

Comparison between occupancy probability and MaxEnt probability of all sampled grid cells revealed a logarithmic trend and parity (S2 Fig). This relationship ascertained reliability of MaxEnt modelled distribution for subsequent use.

### 3.3 Population models

With an effort of 97313 trap nights, we obtained about 0.51 million photographs (which were suitable for species identification). We recorded 26,130 images of snow leopards and identified 126 unique adult individuals with 440 detections which were suitable for population estimation.

The null model considering single landscape session had an average density estimate of 1.21 (CI 0.98 to 1.5) that produced the population estimate of 576 (CI 465–713) (Table 2). The null model considering block-specific session, had varying densities for every block (Fig 4B), and the pooled landscape estimate (without covariate) was 459 (CI 363–580). The covariate-based model considering MaxEnt probability differed from the null model by an AIC of 19 (S4 Table). This covariate model was subsequently used as the most reliable estimate for density (Fig 4B) and population across the landscape (Table 2). The total population estimate based on the covariate model was 477 (CI 380–598). Snow leopard densities varied across the sampling blocks (Fig 4B) and ranged from 0.003 to 3.177 individuals/100 km^2^. Density was highest in Hemis National Park and least in the Changthang High Altitude Cold Desert Wildlife Sanctuary (Table 3). In total, around 39% snow leopards (~185 individuals) had major part of their activity within protected areas. Nonetheless, around 57% snow leopards in the landscape utilized protected areas for their modelled movement (S5 Table).

**Table 2:** Spatially explicit density model. Density parameter estimates used in modelling snow leopard population. $\hat{D}$ is the modelled density, σ is the detection function, g_0_ is the activity centre.

Parameters	Estimates	
Models	D(.) g_0_(.)σ(.)	D(covariate) g_0_(.)σ(.)
$\hat{D}$ SECR (per 100 km^2^)	1.32 (± 0.12)	1.00 (± 0.14)
σ (SE) (km)	4.08 (±0.14)	4.09 (± 0.15)
g_0_ (SE)	0.003 (± 0.0003)	0.003 (± 0.0003)

**Table 3:** Snow leopard population across different regions in Ladakh. Camera trap variables and population parameters of snow leopard in the protected areas and multi-use areas of Ladakh. (D: average density per 100 km^2^, g_0_: detection probability at the activity centre of the animal, σ: spatial scale of detection in km).

Site	Unique Captures	Camera locations	D ± SE	g_0 _± SE	σ ± SE
Changthang Wildlife Sanctuary block 1	7	163	0.264 ± 0.118	0.0032 ± 0.00029	4.916 ± 0.859
Changthang Wildlife Sanctuary block 2	2	62	0.191 ± 0.218	0.0032 ± 0.00029	4.366 ± 1.897
Hemis National Park	59	442	2.073 ± 0.278	0.0032 ± 0.00029	3.699 ± 0.142
Kargil multi-use area	14	55	1.257 ± 0.480	0.0032 ± 0.00029	6.011 ± 1.107
Leh multi-use area	12	60	1.029 ± 0.434	0.0032 ± 0.00029	5.658 ± 1.149
Zanskar multi-use area	6	49	0.398 ± 0.186	0.0032 ± 0.00029	5.219 ± 0.854
Nubra Shyok Wildlife Sanctuary	26	125	0.895 ± 0.211	0.0032 ± 0.00029	5.623 ± 0.575

## 4. Discussion

The study provides the population status of one of the world’s largest snow leopard populations, using the most intensive and extensive systematic sampling and robust analyses to date. We show that the union territory of Ladakh holds ~68% (477 individuals, CI 380–598) of India’s snow leopard population (~709) [53]. While the null model, which is widely used population estimate elsewhere, had higher predicted population, we preferred statistically accurate and ecologically meaningful covariate-based model estimate. The selected model also represented conservative estimate as compared to other models. We captured 26% unique individuals of the estimated population (126 individuals out of estimated 477), by effectively sampling 18% of the total occurrence area (8,604 km^2^ out of 47,572 km^2^). Hence, while the estimated population is four times higher than the captured individuals, it is a conservative estimate of the total habitat occupied by snow leopards in the region. These estimates place Ladakh as having the highest snow leopard densities recorded globally. The global mean density of snow leopards is estimated to range from 0.92 to 1.8 individuals per 100 km² [11], with the previously highest recorded densities reported in Tibet (1.06 to 1.84 individuals per 100 km²) [54] and Sanjiangyuan, China (0.68 to 1.21 individuals per 100 km²) [55]. Our findings surpass these records, with Hemis National Park demonstrating a density of 2.073 ± 0.278 individuals per 100 km², the highest globally documented density over a relatively large area. Additionally, the densities in the multi-use areas of Kargil and Leh are statistically comparable to the previously reported highest densities in Tibet [54]. The high densities of snow leopards in both protected and multi-use areas of Ladakh underscore the global conservation significance of this region. These high densities and the species’ extensive distribution are largely driven by the availability of abundant wild and domestic prey (Fig 3, Table 1), rugged and remote habitat areas, and very low human population in sparsely placed smaller villages, supported by conservation investments, cultural reverence for wildlife among local communities, and traditional pastoral practices. The survival of snow leopards in Ladakh thus hinges on intricate socioecological relationships. We interpret our findings in the context of global methodological advancements introduced by our study and their implications for developing a sustainable model of snow leopard conservation at the local level.

We deployed cameras for photographing the forehead patterns of snow leopard obtaining reliable individual identification process in place of the currently used flank photographs. This approach reduces the camera traps required to half, since only one camera is required as compared to double cameras required to photograph both flanks. While the use of a few old camera trap models may not produce photographs with the necessary clarity of the forehead patterns, the new models have an improved and satisfactory resolution. The study provides India’s first national photo-library of snow leopards, wherein unique pelage pattern of every individual are digitized using a machine learning program. Other range countries and Indian states can contribute to this library to generate a critical tool for monitoring snow leopard poaching and body part trafficking. Over a longer period, this library can be valuable for understanding snow leopard demographics and transboundary movement. The technological outcomes of this study: android applications for occupancy sampling (MSTrIPES), data archiving software, and snow leopard individual identification program, can be used for conceiving a systematic snow-leopard assessment across their range. Catering to the regional conservation requirements and advancing our global comprehension on population estimation and ecology of the elusive snow leopard, the present study extends frontiers.

While global snow leopard population assessment is limited by several challenges and biases, recent initiatives like PAWS offer opportunity to establish scientific protocols for reliable assessment and monitoring. While methods like double-sampling are encouraged, the nuances of conducting robust ground sampling and its quantitative framework are seemingly left for contextual adaptations. This has likely resulted in varied methods and resolutions, and differential scientific reliability. Such variability can hinder population comparison even within a country, where different regions use varying methods. The present study could be upscaled at national level in India to overcome prevalent heterogeneity in study designs and population assessments observed to date. Our study offers a landscape-scale approach for systematizing the double-sampling framework suggested by PAWS. By implementing it in one of the most challenging terrains inhabited by snow leopards and by intensively sampling relatively large areas, our study offers an approach that can be readily integrated as the method for periodic population assessment of snow leopards, their prey and habitat in India and across its global range. The present estimation could be improved by replacing the occurrence of herbivores by their density estimates, and by camera trapping a larger proportion of the habitat to improve on estimate precision of snow leopard population.

While snow leopard densities varied across the region, multiuse areas had a higher proportion of the population as compared to protected areas. This pattern requires cautious interpretation. Protected areas in the region (e.g., Hemis National Park, Nubra Shyok/Karakoram Wildlife Sanctuary and Changthang High Altitude Cold Desert Wildlife Sanctuary) serve to safeguard valuable natural features like the Siachen glacier (world’s second largest non-polar glacier), permafrost regions, cold desert systems, and trans-Himalayan lakes, from land-use changes and anthropogenic impacts. These protected areas are also sparsely used by many local communities that have resided and shaped human-wildlife cultures in the region. These protected areas are inherently low in productivity owing to the cold xeric systems, albite the only regions with rare species like the Tibetan antelope, Tibetan gazelle, and wild yak [27]. The most productive wildlife habitats in the region are taken over for human use [47]. These multi-use areas, particularly along the river Indus, are more productive and are hence historically largely occupied by people with large portions being used for agriculture, pastoralism, settlement, and infrastructure. Our results show these productive multi-use areas constitute the prime habitat for Ladakh urial and blue sheep (Fig 5, S1 Fig), which further supports high snow leopard density in the region (Fig 4). These multi-use productive valleys are the only forage areas during winters, resulting in local elevational migration of both wildlife and domestic stock from high-elevation regions [56,57] increasing the co-occurrence of wildlife, people, and livestock (S3 Fig). This land sharing between snow leopards, wild herbivores, livestock, and people in the multi-use area of Ladakh can thus be seen as an outcome of a seasonal resource restricted landscape. Despite resource driven co-occurrence, snow leopard presence was seen to decline with intensive human settlements (Table 1, Fig 3), indicating that land sharing with snow leopards is parabolic in nature, highest overlap in areas of low use by people. Protection of these multiuse areas (outside of legally protected areas) from increasing human pressures and land use conversions brought by increasing developmental and infrastructure projects is thus necessary for conserving wildlife populations including the snow leopard. A protected area restricted conservation can be unsustainable to protect snow leopards and other wildlife in the long-term, particularly in areas like Ladakh.

Traditional non-intensive land uses, especially transhumance pastoralism - the major occupation of the people, are known to be conducive for snow leopard occurrence. Snow leopards utilize domestic animals, particularly in the resource restricted season [29–31]. Analysis of snow leopard scats from the same region confirmed domestic herbivores including sheep, goat, and cattle to constitute highest proportion of snow leopard diet (Unpublished, Raina et al. ). Resultantly, the occurrence (Table 1, Fig 3) of snow leopards was seen increasing with livestock presence in the region. Livestock depredation by large carnivores in this region has resulted in conflict with people, which in the past was linked with retaliatory killings of carnivores [34]. In the last few decades, conservation investments in form of financial compensation for livestock loss, handholding of communities for benefiting from nature-based (primarily snow leopard) tourism and upholding cultural practices that encourage protection of wildlife has resulted in high population of snow leopards across the landscape. Thus, if traditional pastoral practices are encouraged and supplemented with economic securities, the snow leopard population in the region can extend an inclusive opportunity for the community to benefit from ecotourism and exemplify a sustainable land-sharing model. Snow leopard coexistence can thus be achieved on multiuse land by land-sparing from intensive anthropogenic land-use and land-sharing with traditional land-use, supplemented by community-based ecotourism.

Ongoing changes, including intensive land use conversion, climatic changes and international boundary conflicts are rapidly changing the land use, traditional practices, and human-wildlife interactions (Vision 2050 for Ladakh UT 2020). Climatic changes can cause change in the nutritional forage plants by promoting climate following “range-shifters” that are not always nutritional or conventional food for wild herbivores. Loss of food resources in these xeric conditions can have cascading impacts on the trophic complexities. The twin challenges of changing climate and vegetation, along with increasing human-wildlife interactions can change the disease regime, impacting many wild herbivores in the region [58]. Very little is known about changing diseases and vegetation regimes in the region, particularly due to domestic animals like free ranging dogs, posing a challenge for conservation of snow leopards, wild herbivores, and their habitat [59]. Furthermore, present landscape-scale sampling shows less occurrence of wild herbivores like the blue sheep and the ibex in the western region of Ladakh, despite its habitat suitability and relatively high productivity. This region had a long history of unsustainable wildlife harvest that likely has depleted abundance of wild herbivores and snow leopards [30,60]. Reintroducing and supplementing these species to suitable habitats could be a vital tool for their recovery in the region. Other major threats to snow leopards arise from illegal wildlife trade as its pelt is in high demand, retaliatory killing, and free-ranging dogs [59]. Profits from snow leopard based community ecotourism opportunities has reduced poaching, yet technology-based smart patrolling like MSTrIPES, which is used for tiger protection in India, can be a vital tool for systematic law enforcement and scientific monitoring of wildlife populations [35]. With proactive conservation measures, the landscape has the potential of furthering this as one of the largest populations of snow leopards in the world as well as showcase an exemplary coexistence model.

Considering the global importance of snow leopards as a flagship for many nations, particularly the “third pole” (e.g., the Bishkek declaration), it is imperative to devise reliable and replicable methods of their estimation for periodic monitoring of their populations [10]. Monitoring the changing populations of snow leopards can guide appropriate and timely investments and extend opportunities for ecotourism arising from the snow leopard’s charisma. Systematizing the estimation approach as suggested in the present study can thus benefit the species and the people in the region. It will strengthen the Kunming-Montreal Global Biodiversity Framework targets for 2030 encompassing conservation of the species and minimizing the negative impacts of ongoing changes (Target 1, Target 3, Target 4 and Target 8). For India, systematic population monitoring of snow leopards can significantly contribute to the conservation of this big cat, in pars with the periodic assessments of tiger and leopards, thereby strengthening its leadership at the International Big Cat Alliance. Survival of one of the largest populations of snow leopard in this landscape with a history of armed conflict at international boundaries extends an opportunity to sustainable land-use and an international dialogue to minimize the land-use conversion arising from conflicts. We see systematic transboundary snow leopard monitoring using proposed scientific assessments to be an important step for translating the conservation of snow leopards into broader actions ranging from biodiversity recovery to international peace agreements.

## Supporting information

S1 Table List of variables used. Details on the sources, resolution and scale of remote sensing datasets used.
(DOCX)

S2 Table Correlation matrix. Correlation amongst variables used in various statistical analyses, derived using Pearson’s correlation test.
(DOCX)

S3 Table Comparison of occupancy models. Competing models used for deriving occupancy (Ψ) and detection (p) of snow leopards in Ladakh. The model with least AIC was considered best explanatory model (bold).
(DOCX)

S4 Table SECR model parameters. Competing models for explaining density (D), magnitude (intercept) of detection function (g_0_), and spatial scale of detection function (σ) of snow leopards in Ladakh.
(DOCX)

S5 Table Snow leopard population estimates. Snow leopard population estimates in protected areas and administrative areas of Ladakh. Values in parentheses are confidence intervals.
(DOCX)

S1 Fig MaxEnt response curves. Relationship between species occurrence (y axes) and environmental variables (x axes) as modelled using MaxEnt.
(PNG)

S2 Fig Comparison between occupancy and MaxEnt predictions. Relationship between occurrence probability derived using occupancy model and MaxEnt model within the sampling grids (10 × 10 km) shows high parity with logarithmic trend. It ascertains reliability of MaxEnt based occurrence probability across the landscape, where occupancy could not be evaluated due to absence of sampling.
(PNG)

S3 Fig Overlap between wild and domestic herbivores. Non-linear positive correlation between the encounter rates (sightings per km) of large wild herbivores and domestic herbivores in the Trans-Himalayan region of Ladakh.
(PNG)
