# Supplementary material for: Comprehensive assessment of snow leopard distribution and population in the Indian Trans-Himalaya, Ladakh: Standardizing methods for evidence-based conservation
Source: PLoS One. 2025 May 7;20(5):e0322136. doi: 10.1371/journal.pone.0322136 (PMC12057866; doi:10.1371/journal.pone.0322136)
Supplement: S1 Table — Details on the sources, resolution and scale of remote sensing datasets used. (DOCX) [file pone.0322136.s001.docx]

**S1 Table. List of variables used.** Details on the sources, resolution and scale of remote sensing datasets used.

| **SN** | **Variable, resolution, and source** | **Relevance** |
| --- | --- | --- |
| 1 | **Temperature of the coldest season** 1 km [1] | Movement of animals in the cold xeric system of Ladakh is driven by temperature of the coldest season where animals prefer to migrate to warmer patches. |
| 2 | **Annual precipitation** 1 km [1] | Precipitation in this xeric system is linked with productivity of the area, driving forage availability for herbivores |
| 3 | **Elevation** 30 m [2] | A strong elevational variation in climate and vegetation is observed in the region that determine movement and density of herbivores, which in turn influences carnivores |
| 4 | **Terrain ruggedness** 30 m [3] | The challenging and steep terrain complexities in the region entail high energy expenditure for movement of animals, influencing the resultant occurrence in less rugged areas. |
| 5 | **Slope** 30 m [2] | Animals like the ibex is known to have adapted to utilize steep slope terrain in the region, while others might prefer relatively lesser slope. This can result in terrain determined segregation of herbivore occurrence in the region. |
| 6 | **Distance from valley** 30 m [4] | This can represent escape terrain that the wild animals utilize during threats by competitors, predators, or humans. |
| 7 | **Permanently snow-covered area** 30 m [5] | These regions being devoid of productivity, will attract least animals |
| 8 | **Proximity to water source** 30 m [5] | Water source is a limiting resource in this xeric region governing movement of many animals. |
| 9 | **Moist grassy areas** 30 m [5] | Represent high productive grassy areas, usually along moist regions that can attracts wild and domestic herbivores for forage |
| 10 | **Open natural areas** 30 m [5] | Represent low productive sparse grassy areas across the xeric regions that entail larger movement of animals for procuring food and lesser density |
| 11 | **Enhanced vegetation index** 30 m [5] | Represent the vegetation cover of an area that determines availability of forage for herbivores. |
| 12 | **Human settlement area** 30 m [5] | Smaller settlement areas can positively relate with wild animals due to agricultural/domestic animal utilization by wild herbivores/carnivores, while larger settlements can reduce the occurrence probability. |
| 13 | **Wild herbivore availability** Collected during occupancy sampling across the study region | Wild herbivores like the blue sheep (*Pseudois nayaur*), Asiatic ibex (*Capra ibex sibiricap*), and Ladakh urial (*Ovis vignei vignei*) are the preferred prey of the snow leopard, increasing its density [6]. |
| 14 | **Domestic herbivore availability** Collected during occupancy sampling across the study region | Snow leopards also utilize domestic livestock (domestic yak calves, xo, cattle, ponies, mules, sheep, and goats) in the region increasing their occurrence [7–9]. |
| 15 | **Protected areas** <https://mstripes.wii.gov.in/> | Protected areas usually relate with higher abundance of wild animals |
| 16 | **Land-use cover** [10] | Used to background in maps along with elevation |

**References:**

1. Fick SE, Hijmans RJ. WorldClim 2: new 1-km spatial resolution climate surfaces for global land areas. International Journal of Climatology. 2017;37: 4302–4315. doi:10.1002/joc.5086

2. Gorokhovich Y, Voustianiouk A. Accuracy assessment of the processed SRTM-based elevation data by CGIAR using field data from USA and Thailand and its relation to the terrain characteristics. Remote sensing of Environment. 2006;104: 409–415. Available: https://www.sciencedirect.com/science/article/pii/S0034425706002008?casa_token=MeiKuYpxLM4AAAAA:EaPXhNTwcF5mP5NJrJ3d2uAUaJaCHlbYgg2rrzYFjjmVj2RkNvZhWxpuD8KiboXE-IpcQG6jaw

3. Riley SJ, DeGloria SD, Elliot R. Index that quantifies topographic heterogeneity. intermountain Journal of sciences. 1999;5: 23–27. Available: http://download.osgeo.org/qgis/doc/reference-docs/Terrain_Ruggedness_Index.pdf

4. Köthe R, Bock M. Development and use in practice of SAGA modules for high quality analysis of geodata. FREE AND OPEN GIS-SAGA-GIS. 2006;115: 85–96. Available: https://www.researchgate.net/profile/Juergen-Boehner/publication/326131093_SAGA_-_Analysis_and_Modelling_Applications/links/5b9100cea6fdcce8a4c9a474/SAGA-Analysis-and-Modelling-Applications.pdf#page=89

5. Bhasin A, Dolker P, Raina P, Ghosal S. Land use and land cover change detection using remote sensing in the Trans Himalayan Region of Ladakh, India. ECS Transactions. 2022;107: 2985.

6. Chundawat RS, Qureshi Q. Planning wildlife conservation in Leh and Kargil districts of Ladakh, Jammu & Kashmir. Wildlife Institute of India, Dehradun, India; 1999.

7. Namgail T, Fox JL, Bhatnagar YV. Carnivore-Caused Livestock Mortality in Trans-Himalaya. Environmental Management. 2007;39: 490–496. doi:10.1007/s00267-005-0178-2

8. Suryawanshi KR, Bhatnagar YV, Redpath S, Mishra C. People, predators and perceptions: patterns of livestock depredation by snow leopards and wolves. Journal of Applied Ecology. 2013;50: 550–560. doi:10.1111/1365-2664.12061

9. Sharma RK, Bhatnagar YV, Mishra C. Does livestock benefit or harm snow leopards? Biological Conservation. 2015;190: 8–13. doi:10.1016/j.biocon.2015.04.026

10. Buchhorn M, Smets B, Bertels L, De Roo B, Lesiv M, Tsendbazar N-E, et al. Copernicus Global Land Service: Land Cover 100m: Collection 3 Epoch 2015, Globe. Version V3 01)[Data set]. 2020.
