# Supplementary material for: Comprehensive assessment of snow leopard distribution and population in the Indian Trans-Himalaya, Ladakh: Standardizing methods for evidence-based conservation
Source: PLoS One. 2025 May 7;20(5):e0322136. doi: 10.1371/journal.pone.0322136 (PMC12057866; doi:10.1371/journal.pone.0322136)
Supplement: S2 Table — Correlation amongst variables used in various statistical analyses, derived using Pearson’s correlation test. (DOCX) [file pone.0322136.s002.docx]

**S2 Table. Correlation matrix.** Correlation amongst variables used in various statistical analyses, derived using Pearson’s correlation test.

| **Layer** | **BS** | **DH** | **ELV** | **VEG** | **HS** | **IB** | **KI** | **MG** | **OA** | **PF** | **PPT** | **RUG** | **SLP** | **VLY** | **DW** | **WP** | **WT** |
| --- | --- | --- | --- | --- | --- | --- | --- | --- | --- | --- | --- | --- | --- | --- | --- | --- | --- |
| **BS** | 0 | 0.61 | -0.08 | -0 | -0 | 0.09 | -0 | -0.13 | 0.19 | -0.14 | -0.17 | 0.14 | 0.09 | 0.07 | -0.04 | 0.98 | 0.09 |
| **DH** | 0.61 | 0 | -0.04 | -0 | -0 | 0.49 | -0 | -0.15 | 0.12 | -0.06 | -0.18 | 0.15 | 0.16 | 0.01 | -0.04 | 0.7 | 0.01 |
| **ELV** | -0.08 | -0.04 | 0 | -0.4 | -0.4 | -0.1 | 0.24 | 0.13 | -0.1 | 0.45 | 0.14 | 0.15 | -0.3 | -0.12 | -0.11 | -0.11 | -0.92 |
| **VEG** | -0.02 | -0.02 | -0.41 | 0 | 0.32 | -0.1 | 0.15 | -0.44 | -0.2 | -0.39 | -0.23 | -0.5 | -0.2 | -0.06 | 0.06 | -0.02 | 0.58 |
| **HS** | -0.02 | -0.04 | -0.37 | 0.32 | 0 | -0 | -0.1 | -0.1 | 0.01 | -0.14 | -0.05 | -0.3 | -0.1 | -0.07 | 0.01 | -0.03 | 0.39 |
| **IB** | 0.09 | 0.49 | -0.06 | -0.1 | -0 | 0 | -0.1 | -0.03 | 0.07 | 0.05 | -0.08 | 0.15 | 0.14 | 0.07 | -0.04 | 0.31 | -0.02 |
| **KI** | -0.02 | -0.04 | 0.24 | 0.15 | -0.1 | -0.1 | 0 | -0.09 | -0.1 | -0.08 | 0.05 | -0.3 | -0.3 | -0.05 | 0.07 | -0.04 | -0.09 |
| **MG** | -0.13 | -0.15 | 0.13 | -0.4 | -0.1 | -0 | -0.1 | 0 | -0.3 | 0.14 | 0.91 | 0.21 | 0.07 | 0.08 | -0.05 | -0.14 | -0.37 |
| **OA** | 0.19 | 0.12 | -0.09 | -0.2 | 0.01 | 0.07 | -0.1 | -0.33 | 0 | -0.16 | -0.44 | 0.26 | 0.16 | 0.08 | -0.14 | 0.2 | 0.11 |
| **PF** | -0.14 | -0.06 | 0.45 | -0.4 | -0.1 | 0.05 | -0.1 | 0.14 | -0.2 | 0 | 0.08 | 0.31 | 0.07 | -0.04 | -0.11 | -0.13 | -0.48 |
| **PPT** | -0.17 | -0.18 | 0.14 | -0.2 | -0.1 | -0.1 | 0.05 | 0.91 | -0.4 | 0.08 | 0 | 0.02 | -0.1 | 0.06 | -0.04 | -0.19 | -0.3 |
| **RUG** | 0.14 | 0.15 | 0.15 | -0.5 | -0.3 | 0.15 | -0.3 | 0.21 | 0.26 | 0.31 | 0.02 | 0 | 0.71 | 0.17 | -0.34 | 0.16 | -0.33 |
| **SLP** | 0.09 | 0.16 | -0.3 | -0.2 | -0.1 | 0.14 | -0.3 | 0.07 | 0.16 | 0.07 | -0.06 | 0.71 | 0 | 0.15 | -0.12 | 0.11 | 0.1 |
| **VLY** | 0.07 | 0.01 | -0.12 | -0.1 | -0.1 | 0.07 | -0.1 | 0.08 | 0.08 | -0.04 | 0.06 | 0.17 | 0.15 | 0 | -0.04 | 0.08 | 0.06 |
| **DW** | -0.04 | -0.04 | -0.11 | 0.06 | 0.01 | -0 | 0.07 | -0.05 | -0.1 | -0.11 | -0.04 | -0.3 | -0.1 | -0.04 | 0 | -0.05 | 0.13 |
| **WP** | 0.98 | 0.7 | -0.11 | -0 | -0 | 0.31 | -0 | -0.14 | 0.2 | -0.13 | -0.19 | 0.16 | 0.11 | 0.08 | -0.05 | 0 | 0.11 |
| **WT** | 0.09 | 0.01 | -0.92 | 0.58 | 0.39 | -0 | -0.1 | -0.37 | 0.11 | -0.48 | -0.3 | -0.3 | 0.1 | 0.06 | 0.13 | 0.11 | 0 |

(*Abbreviations*: BS: encounter rate of blue sheep, DH: encounter rate of domestic herbivores, ELV: elevation, VEG: enhance vegetation index, HS: proportion of human settlements, IB: encounter rate of ibex, KI: encounter rate of kiang, MG: proportion of moist grassy areas, OA: proportion of open natural areas, PF: permanently snow-covered areas, PPT: annual precipitation, RUG: terrain ruggedness, SLP: terrain slope, VLY: distance from valley, DW: distance from water, WP: wild prey encounter rate, WT: temperature of coldest season)
