# Supplementary material for: Comprehensive assessment of snow leopard distribution and population in the Indian Trans-Himalaya, Ladakh: Standardizing methods for evidence-based conservation
Source: PLoS One. 2025 May 7;20(5):e0322136. doi: 10.1371/journal.pone.0322136 (PMC12057866; doi:10.1371/journal.pone.0322136)
Supplement: S3 Table — Competing models used for deriving occupancy (Ψ) and detection (p) of snow leopards in Ladakh. The model with least AIC was considered best explanatory model (bold). (DOCX) [file pone.0322136.s003.docx]

**S3 Table.** **Comparison of occupancy models.** Competing models used for deriving occupancy (Ψ) and detection (p) of snow leopards in Ladakh. The model with least AIC was considered best explanatory model (bold).

| Model | AIC | deltaAIC | no.Par. |
| --- | --- | --- | --- |
| Ψ (ele, herb, hs), p(.) | **1648.59** | **0** | **5** |
| Ψ (ele, wp, hs, open), p(.) | 1655.1 | 6.51 | 6 |
| Ψ (ele, wp, hs, open, vall), p(.) | 1657.07 | 8.48 | 7 |
| Ψ (wtemp, wp, hs, open, vall), p(.) | 1669.21 | 20.62 | 7 |
| Ψ (herb), p(.) | 1674.6 | 26.01 | 3 |
| Ψ (herb, open), p(.) | 1676.26 | 27.67 | 4 |
| Ψ (wp), p(.) | 1680.35 | 31.76 | 3 |
| Ψ (ele), p(.) | 1712.23 | 63.64 | 3 |
| Ψ (pf), p(.) | 1717.41 | 68.82 | 3 |
| Ψ (wtemp), p(.) | 1721.36 | 72.77 | 3 |
| Ψ (open), p(.) | 1734.15 | 85.56 | 3 |
| Ψ (hs), p(.) | 1736.26 | 87.67 | 3 |
| Ψ (.), p(.) | 1736.34 | 87.75 | 2 |
| Ψ (rug), p(.) | 1737.47 | 88.88 | 3 |

***Abbreviations****: dom: domestic herbivore encounter rate, ele: elevation, herb: ungulate encounter rate (wild + domestic), hs: human settlement density, open: open grassy area, rug: terrain roughness, vall: distance from valley, Wp: wild large herbivore encounter rate, wtemp: minimum temperature of coldest quarter.*
