# Supplementary material for: Comprehensive assessment of snow leopard distribution and population in the Indian Trans-Himalaya, Ladakh: Standardizing methods for evidence-based conservation
Source: PLoS One. 2025 May 7;20(5):e0322136. doi: 10.1371/journal.pone.0322136 (PMC12057866; doi:10.1371/journal.pone.0322136)
Supplement: S4 Table — Competing models for explaining density (D), magnitude (intercept) of detection function (g0), and spatial scale of detection function (σ) of snow leopards in Ladakh. (DOCX) [file pone.0322136.s004.docx]

**S4 Table.** **SECR model parameters.** Competing models for explaining density (D), magnitude (intercept) of detection function (g_0_), and spatial scale of detection function (σ) of snow leopards in Ladakh.

| Model | Detection | No.Para | AICc | dAICc |
| --- | --- | --- | --- | --- |
| D(habitat suitability) g_0_ (.) σ(.) | Halfnormal | 4 | 3098.87 | 0 |
| D(.) g_0_(.) σ(.) | Halfnormal | 3 | 3118.33 | 19.46 |
