# Supplementary material for: Comprehensive assessment of snow leopard distribution and population in the Indian Trans-Himalaya, Ladakh: Standardizing methods for evidence-based conservation
Source: PLoS One. 2025 May 7;20(5):e0322136. doi: 10.1371/journal.pone.0322136 (PMC12057866; doi:10.1371/journal.pone.0322136)
Supplement: S5 Table — Snow leopard population estimates in protected areas and administrative areas of Ladakh. Values in parentheses are confidence intervals. (DOCX) [file pone.0322136.s005.docx]

**S5 Table.** **Snow leopard population estimates.** Snow leopard population estimates in protected areas and administrative areas of Ladakh. Values in parentheses are confidence intervals.

| Area | | Population estimate | |
| --- | --- | --- | --- |
| *Protection units* | ***Inside  area boundary*** | | ***Using  area*** |
| Hemis National Park | | 56 (40 – 82) | 88 (62 – 128) |
| Changthang High Altitude Cold Desert Wildlife Sanctuary | | 67 (44 – 108) | 94 (61 – 153) |
| Nubra Shyok/Karakoram Wildlife Sanctuary | | 62 (42 – 96) | 89 (61 – 134) |
| Total Protected area | | 185 (126 – 287) | 271 (184 – 415) |
| Multi-use area | | 292 (254 – 311) | |
| *Administrative units* | | |  |
| Leh district | | 350 (291 – 408) | |
| Kargil district | | 127 (89 – 190) | |
| *Total* | | **477 (380 – 598)** | |
