# Supplementary figures and images for: Comprehensive assessment of snow leopard distribution and population in the Indian Trans-Himalaya, Ladakh: Standardizing methods for evidence-based conservation
Source: PLoS One. 2025 May 7;20(5):e0322136. doi: 10.1371/journal.pone.0322136 (PMC12057866; doi:10.1371/journal.pone.0322136)

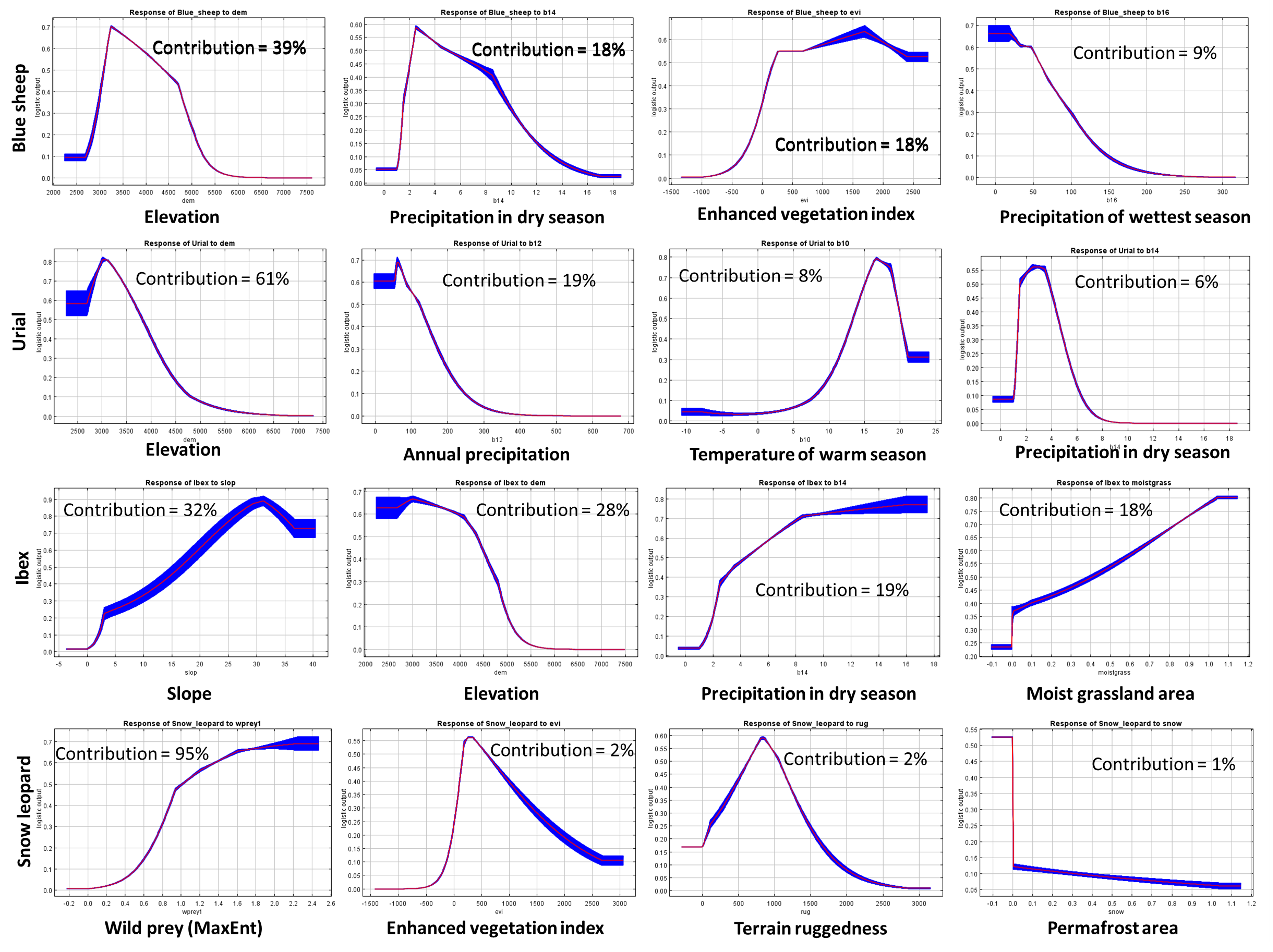

Supplement: S1 Fig — Relationship between species occurrence (y axes) and environmental variables (x axes) as modelled using MaxEnt. (PNG) [file pone.0322136.s006.png]

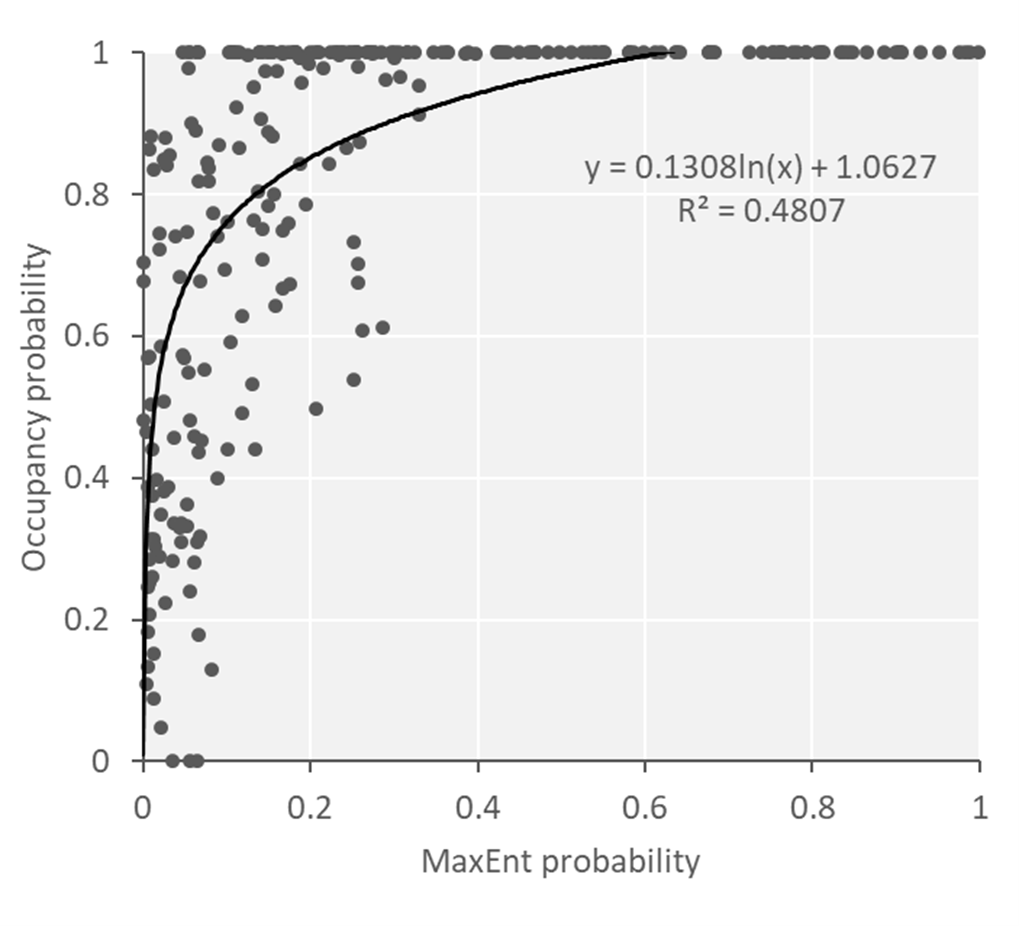

Supplement: S2 Fig — Relationship between occurrence probability derived using occupancy model and MaxEnt model within the sampling grids (10 × 10 km) shows high parity with logarithmic trend. It ascertains reliability of MaxEnt based occurrence probability across the landscape, where occupancy could not be evaluated due to absence of sampling. (PNG) [file pone.0322136.s007.png]

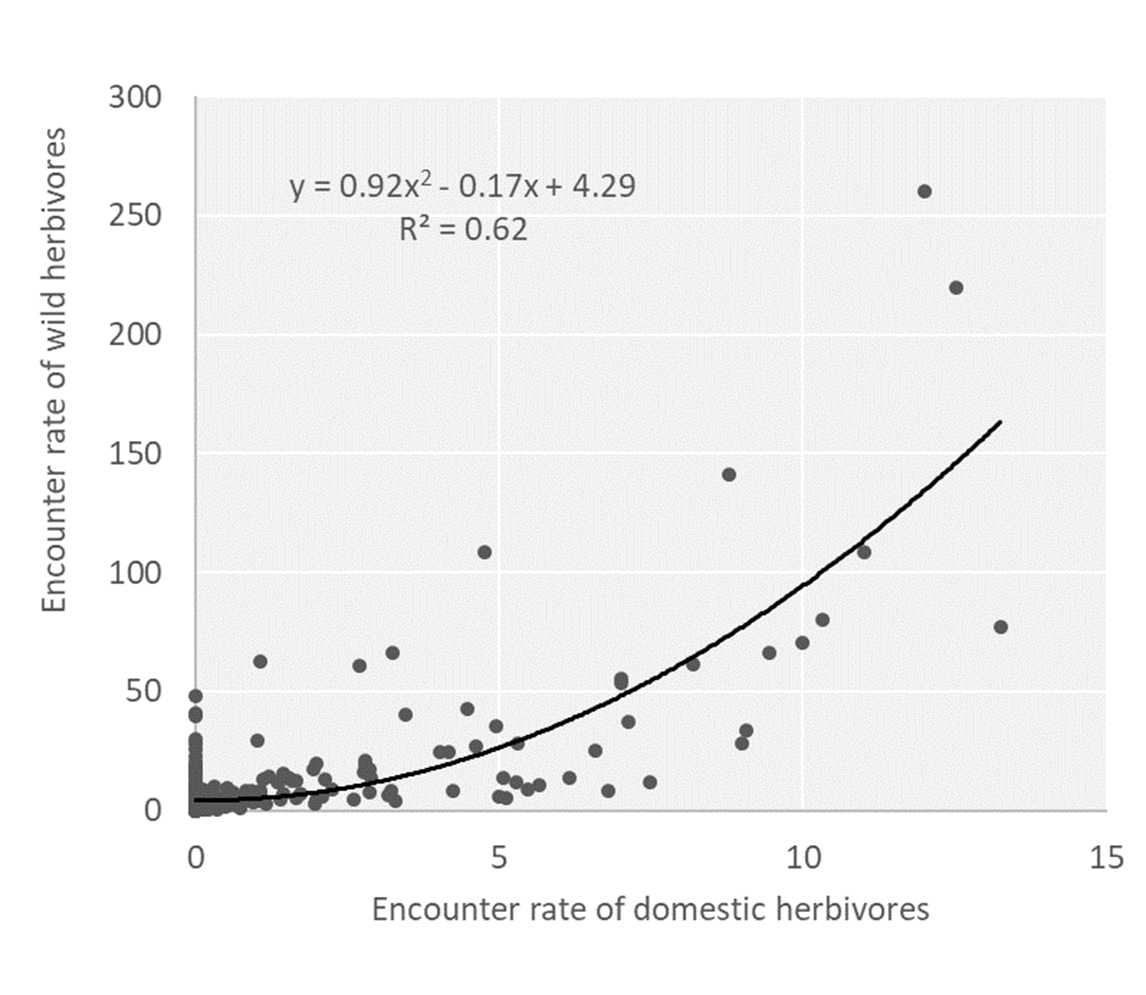

Supplement: S3 Fig — Non-linear positive correlation between the encounter rates (sightings per km) of large wild herbivores and domestic herbivores in the Trans-Himalayan region of Ladakh. (PNG) [file pone.0322136.s008.png]
